# Carfilzomib Mitigates Lipopolysaccharide/D-Galactosamine/Dimethylsulfoxide-Induced Acute Liver Failure in Mice

**DOI:** 10.3390/biomedicines11113098

**Published:** 2023-11-20

**Authors:** Dhafer Y. Alhareth, Abdulrazaq Alanazi, Wael A. Alanazi, Mushtaq A. Ansari, Mahmoud N. Nagi, Sheikh F. Ahmad, Mohamed S. M. Attia, Ahmed Nadeem, Saleh A. Bakheet, Sabry M. Attia

**Affiliations:** Department of Pharmacology and Toxicology, College of Pharmacy, King Saud University, Riyadh 11451, Saudi Arabiasbakheet@ksu.edu.sa (S.A.B.)

**Keywords:** animal model, hepatotoxicity, inflammation, carfilzomib, oxidative and nitrosative stress

## Abstract

Acute liver failure (ALF) is a disease accompanied by severe liver inflammation. No effective therapy is available yet apart from liver transplantation; therefore, developing novel treatments for ALF is urgently required. Inflammatory mediators released by NF-кB activation play an essential role in ALF. Proteasome inhibitors have many medical uses, such as reducing inflammation and NF-кB inhibition, which are believed to account for most of their repurposing effects. This study was undertaken to explore the possible protective effects and the underlying mechanisms of carfilzomib, a proteasome inhibitor, in a mouse model of ALF induced by lipopolysaccharide/D-galactosamine/dimethylsulfoxide (LPS/GalN/DMSO). Carfilzomib dose-dependently protected mice from LPS/GalN/DMSO-induced liver injury, as indicated by the decrease in serum alanine aminotransferase and aspartate aminotransferase levels. LPS/GalN/DMSO increased TNF-α, NF-кB, lipid peroxidation, NO, iNOS, cyclooxygenase-II, myeloperoxidase, and caspase-3 levels. Carfilzomib administration mitigated LPS/GalN/DMSO-induced liver damage by decreasing the elevated levels of TNF-α, NF-кB, lipid peroxidation, nitric oxide, iNOS, cyclooxygenase-II, myeloperoxidase, caspase-3, and histopathological changes. A restored glutathione level was also observed in the carfilzomib-treated LPS/GalN/DMSO mice. Our results demonstrate that carfilzomib protects against LPS/GalN/DMSO-induced ALF by inhibiting NF-кB, decreasing inflammatory mediators, oxidative/nitrosative stress, neutrophil recruitment, and apoptosis, suggesting that carfilzomib may be a potential therapeutic agent for ALF.

## 1. Introduction

Acute liver failure (ALF) is a life-threatening disorder accompanied by severe inflammation of the liver with an increased release of inflammatory mediators [1]. No successful treatment except liver transplantation is available when patients experience ALF. Animal models of ALF provide an excellent tool to understand the pathophysiological mechanisms and to correlate the data with clinical findings. There are several models that are used widely in ALF and lipopolysaccharide/d-galactosamine (LPS/GalN)-mediated ALF is one of the commonly used animal models of ALF [2]. The benefit of this model is that it can generate ALF within a few hours and therefore has become one of the most popular experimental animal models to investigate possible therapeutic agents.

Rodents are comparatively resistant to even high doses of LPS. However, GalN administration sensitizes the rodents to LPS, leading to ALF. The consecutive events in this animal model take place as follows: First, LPS binds to Toll-like receptor 4 on Kupffer cells and triggers the transcriptional stimulation of cytokine genes, particularly tumor necrosis factor alpha (TNF-α) through the activation of pathways that use NF-кB [3]. Second, TNF-α generates different adhesion molecules on sinusoidal endothelial cells in the liver, which are critical for neutrophil extravasation and cytotoxicity [4]. Third, TNF-α has potent neutrophil-activating properties, and the induction of cell death primarily relies on the generation of reactive oxygen species by neutrophils. This process leads to intracellular oxidative stress in the target cells [5]. Fourth, in this model, the induction of apoptosis by TNF-α leads to death of hepatocyte cells [6]. 

The interaction between TNF-α and its receptor on hepatocytes leads to the activation of the NF-кB, which then triggers the expression of genes associated with inflammation and inhibition apoptosis, which facilitates the transmission of survival signals, hence reducing the occurrence of hepatocyte death. However, when administered in large dosages, GalN has the ability to exhaust the cellular uridine triphosphate reserves, and hinder the synthesis of mRNA in hepatocytes and the production of anti-apoptotic proteins in hepatocytes for an extended period of time. The process of transcription inhibits the production of anti-apoptotic genes, hence facilitating the progression of signaling events towards caspase activation and eventual DNA damage. As GalN is only metabolized in liver cells, LPS/GalN induces programed cell death solely in hepatic cells [2]. Previous studies proved the toxic influence of dimethylsulfoxide (DMSO) on hepatic cells in vivo, as demonstrated by the elevated pro-inflammatory cytokines and exacerbating LPS/GalN-inducing ALF, which could afford an improved and useful animal model for ALF and inflammatory hepatic injury [7,8].

Proteasomes are large, multi-subunit protein complexes that degrade cellular ubiquitin-tagged proteins into peptides. Proteasomes regulate the degradation of cellular proteins that control signal transductions, development and cell cycle progressions, antigen processing and immune responses, and inflammation [9]. Proteasome inhibitors are an important class of drugs for the treatment of different types of cancer. They also function as immunosuppressants, inhibit bone resorption, inflammation, and may have other applications [10]. Proteasome inhibition is an antioxidative defense, as it leads to an up-regulation in the gene expression of antioxidative enzymes in hepatocytes [11]. There is an increasing body of data indicating that proteasome inhibitors have a protective effect against oxidative stress in several organs, including the liver, brain, heart, kidney, and in cases of ischemia-reperfusion damage [11,12,13,14,15,16]. 

Carfilzomib (CFZ) is an irreversible inhibitor of the proteasome and is frequently used in the therapeutic management of multiple myeloma [10]. The effects of proteasome inhibition on drug-induced liver damage vary depending on the specific drugs used. Bortezomib and CFZ, but not MG132, show significant efficacy in mitigating acetaminophen-induced liver damage in animal models [16,17]. Proteasome inhibitors were shown to exert anti-inflammatory effects through different mechanisms [18]. NF-кB is a transcriptional regulator of genes involved in inflammatory responses and innate/adaptive immunity, and attention has focused on its pathophysiological role in the diseased liver [19]. NF-κB is rapidly produced in response to oxidative stress and drug-induced liver injury. NF-кB activation stimulates the transcription of genes encoding inflammatory mediators. Due to the action of NF-κB, it has been suggested as a target of the therapeutic pathway for liver injury, and NF-кB inhibition is believed to account for most of the protective effects of proteasome inhibitors [20,21]. We hypothesized that CFZ could prevent NF-кB activation and related inflammatory mediators, reduce hepatic inflammatory stress, and decrease hepatic cell death. To test our hypotheses, we designed a mouse experiment to examine the impact of CFZ on LPS/GalN/DMSO-induced ALF. Moreover, the mechanisms underlying these effects were elucidated.

## 2. Materials and Methods

### 2.1. Animals

Swiss albino mice aged 6–8 weeks and weighing 20–25 g were obtained from the Experimental Animal Care Center of the College of Pharmacy, King Saud University, Riyadh, Saudi Arabia. All toxicity experiments on mice were carried out according to the Guidelines of the Animal Care and Use Committee at King Saud University, Saudi Arabia (KSUSE-2127), and all animals received care and use consistent with the National Institutes of Health (NIH) guidelines. They were housed under conventional laboratory conditions of temperature (23 ± 2 °C), 12 h of light–dark periods, and humidity (50 ± 10%). The mice were fed a standard animal pellet diet and allowed free access to tap water. Both the treatment group and the control group consisted of six animals. All animals were fasted for 18 h before the experiments.

### 2.2. Experimental Design

Carfilzomib (CFZ; Active Biochem, Maplewood, NJ, USA) was dissolved in DMSO and administered intraperitoneally 1 h after LPS/GalN/DMSO (Sigma-Aldrich, St. Louis, MO, USA) model. The final concentration of the CFZ solvent (DMSO) did not exceed 10 µL/kg which does not affect the model LPS/GalN/DMSO hepatotoxicity, as reported earlier [8]. LPS and GalN were dissolved in saline and administered intraperitoneally with DMSO directly after preparations at a dose of (10 µg/kg/400 mg/kg/200 µL/kg). To evaluate the effect of different doses of CFZ, animals were divided randomly into six groups. The first group represented a control group and received saline; the second group received CFZ (2 mg/kg); the third group received LPS/GalN/DMSO (10 µg/kg/400 mg/kg/200 µL/kg); the fourth group received LPS/GalN/DMSO/CFZ (10 µg/kg/400 mg/kg/200 µL/kg/0.5 mg/kg); the fifth group received LPS/GalN/DMSO/CFZ (10 µg/kg/400 mg/kg/200 µL/kg/1 mg/kg); and the sixth group received LPS/GalN/DMSO/CFZ (10 µg/kg/400 mg/kg/200 µL/kg/2 mg/kg). Animals were sacrificed 6 h after injection and sera were separated. 

To explore the mechanism of protection of CFZ on LPS/GalN/DMSO-induced ALF, mice were randomly allocated into 4 groups, each consisting of 12 animals. The first group represented a control group and received saline; the second group received CFZ (2 mg/kg); the third group received a LPS/GalN/DMSO (10 µg/kg/400 mg/kg/200 µL/kg); and the fourth group received LPS/GalN/DMSO/CFZ (10 µg/kg/400 mg/kg/200 µL/kg/2 mg/kg). Each group were subdivided into 2 subgroups each consisting of 6 animals and were sacrificed 2 and 6 h after injection. It should be noted that the 2 hour groups were used to determine TNF-α and NF-кB. 

### 2.3. Serum Separation and Liver Preparations

Blood samples were collected from the heart, under anesthesia, and sera were separated. Mice were sacrificed by cervical dislocation and the livers were isolated and processed for histopathological investigations or quickly frozen in liquid nitrogen and stored at −80 °C until use. 

### 2.4. Biochemical Analysis

Serum alanine aminotransferase (ALT, cat # AL146) and aspartate aminotransferase (AST, cat # AS3804) activities were determined using ELISA detection kits (Randox laboratories Ltd., Crumlin, UK), following the manufacturer’s instructions. Hepatic lipid peroxidation was measured by the thiobarbituric acid reactive substance method [22], using a malondialdehyde (MDA) detection kit (Cayman Chemical, Ann Arbor, MI, USA; cat # 10009055). Hepatic glutathione (GSH) was evaluated using 5,5′-dithiobis(2-nitrobenzoic acid) according to the previously described protocol [23]. Nitric oxide is a marker of nitrosative stress and was assayed in liver homogenates by Griess reagent [24] using a nitrate/nitrite detection kit (Cayman Chemical, Ann Arbor, MI, USA; cat # 780001) following the manufacturer’s guidelines. Levels of TNF-α in serum were measured using ELISA kits (EMD Millipore, Gibbstown, NJ, USA; cat # EZMTNFA) according to the manufacturer’s guidelines. Hepatic myeloperoxidase activity (MPO), a marker of neutrophil accumulation, was evaluated according to the previously described protocol [25].

### 2.5. Gene Expression

The liver tissues were kept in RNAlater^®^ (Thermo Fisher Scientific, Waltham, MA, USA; cat # AM7020) for a few weeks at 4 °C before being used. Total cellular RNA from liver tissues was isolated using TRIzol™ Reagent (Thermo Fisher Scientific, Waltham, MA, USA; cat # 15596026) according to the manufacturer’s instructions. A NanoDrop 8000 spectrophotometer (Thermo Fisher Scientific, Waltham, MA, USA) was used to measure the concentration and quality of the isolated RNA at the optical density of 260 nm and the 260/280 ratio, respectively. Only samples with a ratio between 1.8 and 2.1 were used. Moreover, RNA integrity was checked by electrophoresis on a 1% agarose gel. Thereafter, first strand cDNA synthesis was performed using the High-Capacity cDNA Reverse Transcription Kit (Applied Biosystems™, Fisher Scientific, Nepean, ON, Canada; cat # 4368814), based on the manufacturer’s guidelines [26]. Quantitative analysis of specific mRNA expression was performed by real-time reverse transcription–polymerase chain reaction (RT-PCR) by subjecting the resulting cDNA to PCR amplification using 96-well optical reaction plates in a 7500 Fast RT-PCR (Applied Biosystems) [27]. Table 1 lists the primers used in this investigation. The RT-PCR data were analyzed using the relative gene expression method [28]. Results are presented as the fold of changes normalized to the reference gene (GAPDH). It is, however, worth noting that only one reference gene was used in the current study. Thus, there are limitations in terms of RT-PCR accuracy. Therefore, it is necessary to use appropriate combinations of three or more reference genes in future studies to improve RT-PCR accuracy.

### 2.6. Western Blot

Western blot analysis was performed to determine the protein levels of caspase 3 and NF-κB. Liver tissues were homogenized in RIPA lysis buffer (Thermo Scientific^TM^; cat # 89901) and the total protein was determined by BCA protein assay kits (Santa Cruz Biotechnology, Inc., Dallas, TX, USA; cat # sc-202389), following the manufacturer’s instructions. The marker (Bio-Rad Laboratories, Hercules, CA, USA; cat # 1610373) and 30 µg of extracted protein from each sample were separated using gel electrophoresis by 10% sodium dodecyl sulfate–polyacrylamide gel (SDS-PAGE) in a 1X running buffer (Bio-Rad Laboratories, Hercules, CA, USA; cat # 1610734), at a constant voltage of 60 for 30 min followed by 120 voltages for about 2 h [29]. The separated proteins were electrophoretically (25 V, 150 mA for 1 h) transferred to methanol pre-soaking polyvinylidene difluoride (PVDF) membranes (Thermo Fisher Scientific, Waltham, MA, USA; cat # 88518), in a transfer buffer including 25 mM Tris HCl, 192 mM glycine, and 25% methanol. 

Blots were then incubated with diluted (1:5000) primary antibodies against NF-κB (cat # sc-8008), caspase 3 (cat # sc-56052), and β-actin (cat # sc-47778) housekeeping genes (Santa Cruz Biotechnology, Inc., USA), at 4 °C overnight, followed by incubation with peroxidase-conjugated secondary antibodies (Santa Cruz Biotechnology, Inc., Dallas, TX, USA; cat # sc-516102) for 1 h at room temperature. Protein bands were visualized using detection system (EMD Millipore, Gibbstown, NJ, USA; cat # WBLUR0500), according to the manufacturer’s guidelines and as previously described [30]. The immunoreactive bands were quantified densitometrically and were normalized against β-actin, a control for protein loading.

### 2.7. Histopathology

Liver specimens were extracted and fixed in 10% neutral buffered formalin. The hepatic tissue was then sliced, and sections were taken for histopathological assessment. After routine automated processing, the slides were stained using the hematoxylin and eosin stain. An experienced histopathologist then examined and scored the stained slides under light microscopy. For quantitative comparisons of the structural alterations, the abnormalities in the hepatic sections were graded from 0 (normal structures) to 3 (severe pathological changes).

### 2.8. Statistical Analysis

Data were expressed as means ± SD for quantitative measures using Graph Pad Prism 9 software (San Diego, CA, USA). The sample size in the current study was determined based on our experiences in this experiment. With six mice per treatment group and a significance level of 0.05 (two-sided), a statistical power of 80% was needed to detect whether the difference between treatment groups can be achieved [31]. The obtained data were first analyzed for normality and homogeneity of the variables using the Kolmogorov–Smirnov and Bartlett’s tests, respectively. Then, statistical comparisons between different groups were performed using the parametric ANOVA test, followed by Tukey–Kramer multiple comparisons, or the non-parametric Kruskal–Wallis test followed by Dunn’s multiple comparisons.

## 3. Results

### 3.1. CFZ Decreases the LPS/GalN/DMSO-Induced Increase in ALT and AST in Serum

**Figure 1:**
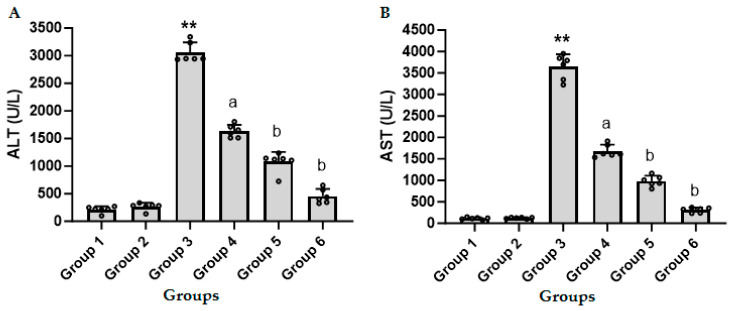
Serum levels of alanine aminotransferase (ALT, (**A**)) and aspartate aminotransferase (AST, (**B**)) in treated and untreated mice (mean ± SD, N = 6). Group 1, control animals; Group 2, CFZ (2 mg/kg); Group 3, LPS/GalN/DMSO (10 µg/kg/400 mg/kg/200 µL/kg); Group 4, LPS/GalN/DMSO/CFZ (10 µg/kg/400 mg/kg/200 µL/kg/0.5 mg/kg); Group 5, LPS/GalN/DMSO/CFZ (10 µg/kg/400 mg/kg/200 µL/kg/1 mg/kg); Group 6, LPS/GalN/DMSO/CFZ (10 µg/kg/400 mg/kg/200 µL/kg/2 mg/kg). ** *p* < 0.01 vs. control group and ^a^ *p* < 0.05, ^b^ *p* < 0.01 vs. LPS/GalN/DMSO group (ANOVA test followed by Tukey–Kramer multiple comparisons test).

Figure 1A,B show the effect of different doses of CFZ on serum ALT and AST activities in LPS/GalN/DMSO-treated mice. Administration of LPS/GalN/DMSO resulted in a significant increase in the level of ALT and AST activities as compared to the control group. Administration of CFZ (0.5, 1.0, and 2 mg/kg) 1 h after LPS/GalN/DMSO significantly decreased ALT and AST in a dose-dependent manner. Maximum protection was obtained with 2 mg/kg of CFZ. CFZ alone showed non-significant changes in serum ALT and AST activity as compared to the corresponding control group.

### 3.2. CFZ Decreases the LPS/GalN/DMSO-Induced Increase in Serum TNF-α, Hepatic NF-кB and Hepatic Caspase 3 Activity

**Figure 2:**
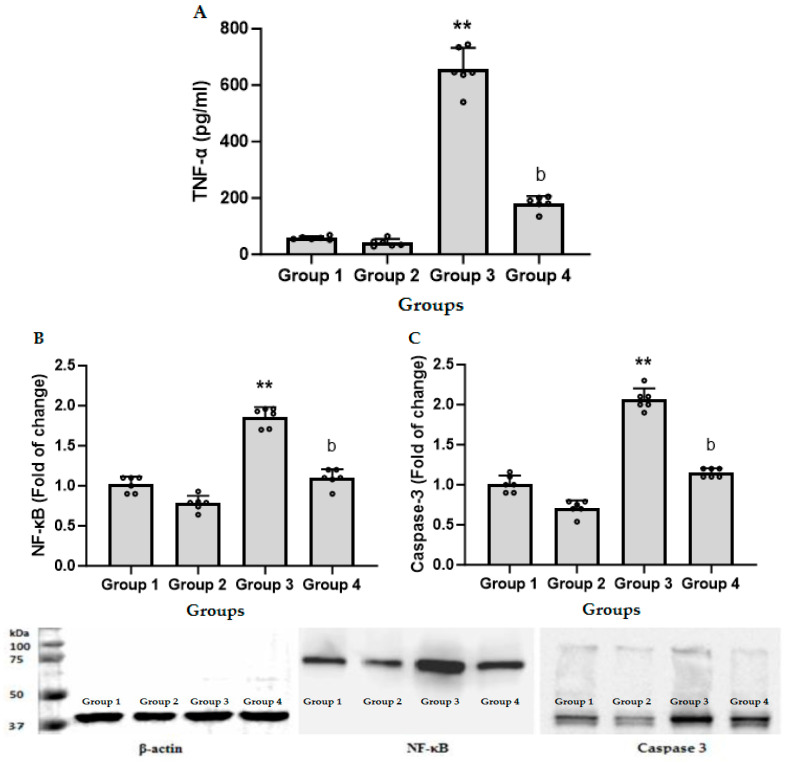
Levels of serum tumor necrosis factor alpha (TNF-α, (**A**)), hepatic nuclear factor kappa beta (NF-кB, (**B**)), and hepatic caspase 3 activation (**C**) in treated and untreated mice (mean ± SD, N = 6). Group 1, control animals; Group 2, CFZ (2 mg/kg); Group 3, LPS/GalN/DMSO (10 µg/kg/400 mg/kg/200 µL/kg); Group 4, LPS/GalN/DMSO/CFZ (10 µg/kg/400 mg/kg/200 µL/kg/2 mg/kg). ** *p* < 0.01 vs. control group and ^b^ *p* < 0.01 vs. LPS/GalN/DMSO group (ANOVA test followed by Tukey–Kramer multiple comparisons test).

In Figure 2A, it is shown that LPS/GalN/DMSO resulted in a significant increase in TNF-α as compared to control group and administration of CFZ (2 mg/kg) together with LPS/GalN/DMSO showed a significant decrease in TNF-α in serum. Figure 2B shows the effect of CFZ on hepatic NF-кB content in LPS/GalN/DMSO-treated and untreated mice. Administration of LPS/GalN/DMSO resulted in a significant increase in the level of NF-кB content as compared to the control group. Administration of CFZ (2 mg/kg) 1 h after LPS/GalN/DMSO showed a significant decrease in NF-кB content. Administration of CFZ alone showed non-significant changes in serum TNF-α and nuclear NF-кB content as compared to the corresponding control group.

Figure 2C shows that administration of LPS/GalN/DMSO resulted in a significant increase in the level of caspase 3 as compared to the control group. Administration of CFZ (2 mg/kg) 1 h after LPS/GalN/DMSO showed a significant decrease in LPS/GalN/DMSO-induced increase in the level of hepatic caspase 3, while CFZ (2 mg/kg) alone showed non-significant changes in the level of caspase 3 as compared to the control group.

### 3.3. CFZ Decreases the LPS/GalN/DMSO-Induced Increase in Hepatic COX-II

**Figure 3:**
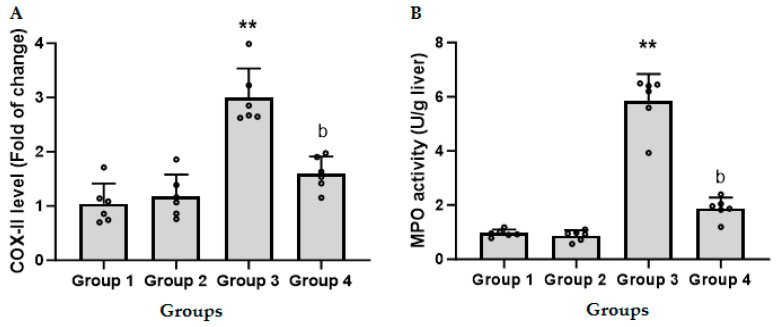
Levels of hepatic cyclooxygenase-II (COX-II, (**A**)) expression and hepatic myeloperoxidase activity (MPO, (**B**)) in treated and untreated mice (mean ± SD, N = 6). Group 1, control animals; Group 2, CFZ (2 mg/kg); Group 3, LPS/GalN/DMSO (10 µg/kg/400 mg/kg/200 µL/kg); Group 4, LPS/GalN/DMSO/CFZ (10 µg/kg/400 mg/kg/200 µL/kg/2 mg/kg). ** *p* < 0.01 vs. control group and ^b^ *p* < 0.01 vs. LPS/GalN/DMSO group (ANOVA test followed by Tukey–Kramer multiple comparisons test).

Figure 3A shows that administration of LPS/GalN/DMSO resulted in a threefold increase in the level of COX-II as compared to the control group. Administration of CFZ (2 mg/kg) 1 h after LPS/GalN/DMSO showed a significant decrease in LPS/GalN/DMSO-induced COX-II. CFZ alone showed a non-significant change in COX-II as compared to the control group.

### 3.4. CFZ Ameliorates the LPS/GalN/DMSO-Induced Neutrophil Recruitment

As presented in Figure 3B, mice administered 2 mg/kg CFZ did not induce any alteration in MPO activity, a marker of neutrophil infiltration, compared with control mice. The LPS/GalN/DMSO injection significantly increased MPO activity in animal hepatic cells compared to the control group. Injection of CFZ (2 mg/kg) 1 h after LPS/GalN/DMSO showed a significant decrease in LPS/GalN/DMSO-induced neutrophil recruitment. CFZ alone showed a non-significant change in neutrophil recruitment as compared to the control group.

### 3.5. CFZ Ameliorates the LPS/GalN/DMSO-Induced Altered Nitrosative and Oxidative Stress

**Figure 4:**
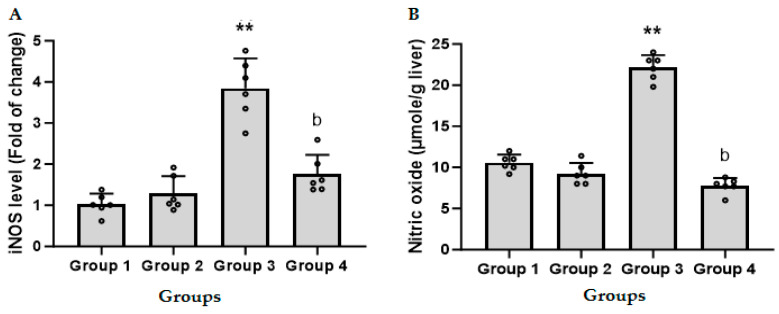
Levels of hepatic inducible nitric oxide synthase (iNOS, (**A**)) and nitric oxide (NO, (**B**)) in treated and untreated mice (mean ± SD, N = 6). Group 1, control animals; Group 2, CFZ (2 mg/kg); Group 3, LPS/GalN/DMSO (10 µg/kg/400 mg/kg/200 µL/kg); Group 4, LPS/GalN/DMSO/CFZ (10 µg/kg/400 mg/kg/200 µL/kg/2 mg/kg). ** *p* < 0.01 vs. control group and ^b^ *p* < 0.01 vs. LPS/GalN/DMSO group (ANOVA test followed by Tukey–Kramer multiple comparisons test).

The effect of CFZ (2 mg/kg) on LPS/GalN/DMSO-induced nitrosative and oxidative stress was evaluated by assessing the levels of hepatic iNOS, nitric oxide, MDA, and GSH. Figure 4A shows that administration of LPS/GalN/DMSO resulted in a four-fold increase in the level of iNOS as compared to the control group. Injection of CFZ (2 mg/kg) 1 h after LPS/GalN/DMSO showed a significant decrease in LPS/GalN/DMSO-induced iNOS expression. CFZ alone showed a non-significant change in iNOS level as compared to the control group.

Administration of LPS/GalN/DMSO resulted in a significant increase in the level of hepatic NO (nitrate and nitrite) as compared to the control group. Injection of CFZ (2 mg/kg) 1 h after LPS/GalN/DMSO showed a significant decrease in nitrate and nitrite (Figure 4B). Administration of CFZ (2 mg/kg) alone showed a non-significant change in nitric oxide level as compared to the control group.

**Figure 5:**
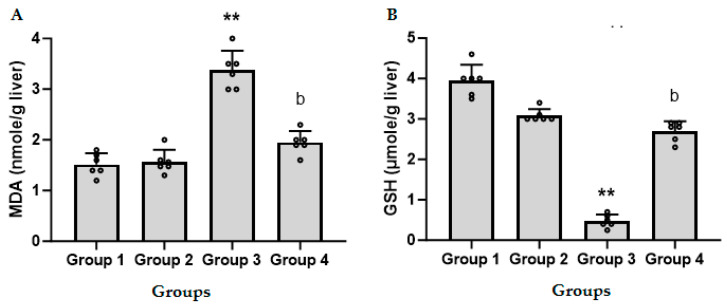
Levels of hepatic malondialdehyde (MDA, (**A**)) and glutathione (GSH, (**B**)) in treated and untreated mice (mean ± SD, N = 6). Group 1, control animals; Group 2, CFZ (2 mg/kg); Group 3, LPS/GalN/DMSO (10 µg/kg/400 mg/kg/200 µL/kg); Group 4, LPS/GalN/DMSO/CFZ (10 µg/kg/400 mg/kg/200 µL/kg/2 mg/kg). ** *p* < 0.01 vs. control group and ^b^ *p* < 0.01 vs. LPS/GalN/DMSO group (ANOVA test followed by Tukey–Kramer multiple comparisons test).

Figure 5A shows the effect of CFZ (2 mg/kg) on the level of MDA, the end product of lipid peroxidation, in LPS/GalN/DMSO-treated and untreated mice. Administration of LPS/GalN/DMSO resulted in a significant increase in the level of MDA as compared to the control group. Administration of CFZ (2 mg/kg) 1 h after LPS/GalN/DMSO showed a significant decrease in LPS/GalN/DMSO-induced lipid peroxidation while CFZ alone showed non-significant changes in lipid peroxidation as compared to the control group.

As shown in Figure 5B, administration of LPS/GalN/DMSO resulted in a significant decrease in GSH level as compared to the control group. Administration of CFZ (2 mg/kg) 1 h after LPS/GalN/DMSO showed a significant increase in GSH. Administration of CFZ (2 mg/kg) alone showed non-significant changes in the GSH level as compared to the control group.

### 3.6. CFZ Decreases LPS/GalN/DMSO-Caused-Histopathological Changes

**Figure 6:**
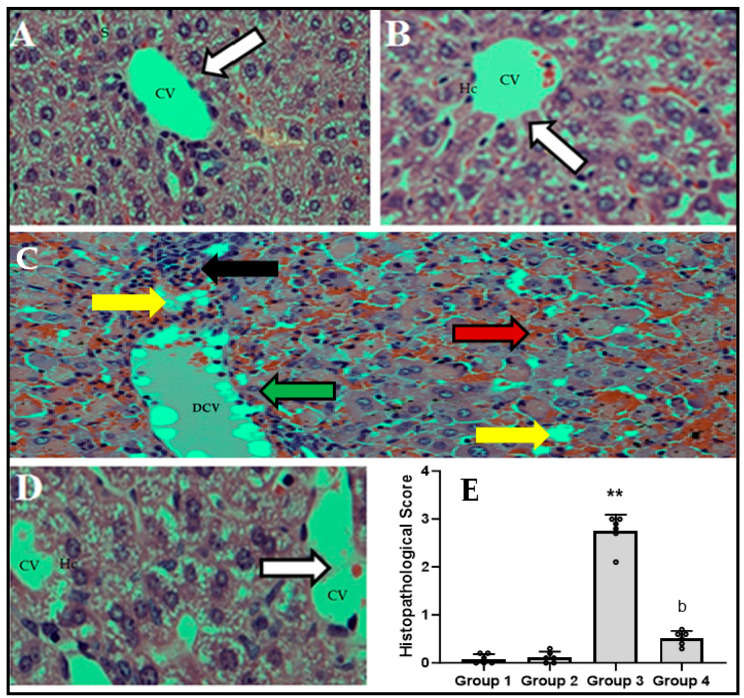
Histopathological investigation of hematoxylin/eosin-stained hepatic sections of treated and untreated mice at a scale bar of 50 µm. (**A**): Liver tissue from control mice showing a normal central vein (CV, white arrow), from which radiate the hepatic cords separated by hepatic sinusoids (S). (**B**): Liver tissue from mice injected with CFZ (2 mg/kg) showing a normal central vein (CV, white arrow) with surrounding intact hepatocytes (Hc). (**C**): Liver tissue from mice injected with LPS/GALN/DMSO (10 µg/kg/400 mg/kg/200 µL/kg) showing infiltration of leukocytes in liver tissue, congestion/dilation in the central vein (DCV) (green arrow) with loss of hepatic architecture, blood sinusoids (red arrow) in some areas, fatty changes (yellow arrow), and foci of hepatic cell necrosis with Councilman body formation (black arrow). (**D**): Liver tissue from mice injected with LPS/GALN/DMSO (10 µg/kg/400 mg/kg/200 µL/kg) and CFZ (2 mg/kg) showing normal central vein (CV, white arrow) and preservation of cellular architecture and integrity of hepatocytes (Hc). The histopathological scoring of the observed pathological changes is shown by the bar graph ((**E**), mean ± SD). ** *p* < 0.01 vs. control group and ^b^ *p* < 0.01 vs. LPS/GalN/DMSO group (Kruskal–Wallis test followed by Dunn’s multiple comparisons test).

Figure 6A–E show the effect of the LPS/GalN/DMSO injection on the histopathological alterations in CFZ (2 mg/kg)-treated and untreated animals. As shown in Figure 6C, administration of LPS/GalN/DMSO resulted in central hepatic vein congestion/dilation, necrosis, blood sinusoids, marked apoptosis, fatty changes, prominent cholestasis, and loss of hepatic architecture as compared to the control animals. Administration of CFZ (2 mg/kg) 1 h after LPS/GalN/DMSO was able to substantially prevent the induced histopathological changes and preserve cellular integrity and architecture (Figure 6D). The increased histopathological score in the LPS/GalN/DMSO-treated mice livers was significantly decreased after CFZ administration (Figure 6E). Additionally, administration of CFZ (2 mg/kg) alone (Figure 6B,E) showed non-significant changes in liver histopathology as compared to the control group.

## 4. Discussion

The present investigation demonstrated that the injection of LPS/GalN/DMSO led to a significant elevation in the activities of ALT and AST, consistent with the observations reported in prior studies [8,32]. The administration of various doses of CFZ one hour after the injection of LPS/GalN/DMSO resulted in a considerable reduction in ALT and AST levels in a dose-dependent manner. The highest level of protection was observed with a dosage of 2 mg/kg of CFZ, thereby confirming the protective properties of CFZ. Subsequently, we investigated the potential mechanism(s) through which CFZ afforded protection to hepatocytes against ALF induced by LPS/GalN/DMSO. Post-treatment with CFZ resulted in a reduction in TNF-α production and the inhibition of NF-κB activation. In addition, CFZ exhibited a reduction in hepatocyte necrosis and apoptosis, as well as a mitigation of oxidative and nitrosative stress. Moreover, CFZ showed a decrease in neutrophil infiltration and a reduction in inflammatory mediators. The protective effect of CFZ was also confirmed by reducing the histopathological changes induced by LPS/GalN/DMSO administration, suggesting that CFZ may be a potential therapeutic agent for ALF.

CFZ exhibited a dose-dependent protective effect against liver injury generated by LPS/GalN/DMSO in mice. This was evidenced by a significant decrease in the elevated levels of serum ALT and AST, which are recognized indicators of liver injury. The aforementioned impact was further investigated in order to elucidate its underlying mechanism of mitigation. The findings of our study agree with previous research, indicating a significant elevation in serum TNF-α levels among mice treated with LPS/GalN/DMSO [8,33,34]. The administration of CFZ following treatment with LPS/GalN/DMSO resulted in a considerable reduction in the increased levels of TNF-α as compared to the group treated with LPS/GalN/DMSO alone. Consequently, CFZ exhibited a preventive effect on liver injury by inhibiting the secretion of TNF-α. 

It is known that TNF-α secretion induced by LPS is regulated by NF-κB in Kupffer cells [35]. Moreover, numerous investigations have revealed that NF-κB plays a vital role in LPS/GalN/DMSO liver injury [8,21,36]. In the state of quiescent Kupffer cells, the NF-κB family members are confined inside the cytoplasm due to their association with inhibitors from the IκB family. Following cellular activation, IκB proteins undergo phosphorylation and subsequent degradation mediated by the proteasome. The activation of NF-кB pathway is contingent upon the functionality of the ubiquitin–proteasome system. Following this, the NF-κB protein has the ability to translocate into the nucleus and bind with DNA, facilitating the transcriptional activation of genes that encode various inflammatory mediators, including iNOS, TNF-α, and COX-II [36,37]. 

In order to further investigate the impact of CFZ on liver damage induced by LPS/GalN/DMSO, the quantification of NF-κB was conducted by Western blotting analysis. The findings of the present study demonstrated that the protein level of NF-κB was suppressed with the administration of CFZ in mice treated with LPS/GalN/DMSO. Therefore, the reduction in TNF-α synthesis by hepatic cells might potentially be linked to the suppression of NF-κB mediated by CFZ. TNF-α generated by LPS-induced Kupffer cells can cause hepatocyte death or survival, depending on the signaling complex bound to the receptor. LPS stimulates TNF-α, which binds to TNF-R and activates the NF-κB pathway. When the NF-κB pathway is activated, a number of anti-apoptotic molecules are up-regulated, providing a survival signal with very low levels of hepatocyte death. TNF-R-mediated overexpression of anti-apoptotic molecules via the NF-κB pathway will not occur in animals challenged with LPS and treated with GalN because GalN inhibits NF-κB activation. In the absence of NF-κB activity, a different complex forms in the cytosol, leading to caspase activation and hepatocyte death [38,39]. Hepatocytes are well known to be particularly susceptible to NF-κB inhibition, which can make them extremely vulnerable to the cytotoxic effects of TNF-α. Caspase 3 expression was evaluated to assess the influence of CFZ on apoptotic activity. LPS/GalN/DMSO substantially increased caspase 3 expression, but CFZ post-treatment decreased caspase 3 expression. In agreement with our data, caspase 3 inhibitors protect mice from LPS/GalN-induced ALF [34,40,41,42,43]. The mechanism of CFZ’s anti-apoptotic effect may be related to its ability to decrease TNF-α secretion.

The increase in neutrophils in tissue is a hallmark of inflammation. MPO provides a quantitative indicator of the tissue’s neutrophil content, and TNF-α promotes neutrophil transmigration in ALF caused by LPS/GalN and plays a role in hepatocyte necrosis [4]. Infiltrated neutrophils induce oxidative hepatocyte death by triggering neutrophil respiratory bursts and degranulation, resulting in widespread hepatocyte necrosis. LPS/GalN/DMSO significantly increased MPO activity in liver tissue, but CFZ treatments considerably decreased MPO activity, indicating less hepatic neutrophil infiltration. The inducible form of the enzyme COX-II is used for the synthesis of prostaglandins, making it a promising target for therapeutic intervention in the context of inflammatory disorders [44]. The regulation of COX-II has been observed to occur throughout the course of inflammation through the NF-κB signaling pathway. The findings of our study indicate that the injection of LPS/GalN/DMSO resulted in an increase in the expression of hepatic COX-II. However, treatment with CFZ was shown to decrease the hepatic expression of COX-II, consequently providing a mitigating effect on hepatic inflammation and damage.

Nitrosative and oxidative stress are caused by redox imbalances, which are often caused by high reactive oxygen and nitrogen species and both types of stress are associated with liver damage [45]. iNOS is a crucial enzyme that generates NO from the amino acid L-arginine and is known to be controlled by the NF-κB signaling pathway during inflammatory development [46]. The role of iNOS activation or overexpression in the pathophysiology of LPS causing an inflammatory disease has received an abundance of attention [47]. Furthermore, inhibiting iNOS could prevent nitrosative stress, which includes NO products in ALF [48]. As a result, we looked at how CFZ affected iNOS and its product NO in liver homogenate. In our investigation, the LPS/GalN/DMSO-treated group demonstrated an increase in hepatic iNOS, which is consistent with prior findings [8,46,49]. CFZ downregulated iNOS expression, reducing hepatic inflammation and injury. 

The NO generated by iNOS is important in the development of LPS/GalN/DMSO-caused inflammation. Following LPS/GalN/DMSO exposure, several cells may participate in NO generation in the liver. At 6 h after LPS/GalN/DMSO treatment, there was a significant increase in hepatic nitrate plus nitrite. In accordance with earlier research, our findings clearly indicate that nitric oxide plays a role in the pathophysiology of LPS/GalN/DMSO-induced hepatotoxicity [46]. The mice treated with CFZ generated much less nitrate and nitrite than the animals treated with LPS/GalN/DMSO alone. These data suggested that NO generation was significantly reduced as a result of CFZ-induced iNOS downregulation.

When the antioxidant defense system is overburdened, it causes lipid peroxidation, resulting in liver injury [49,50]. The formation of free radicals was linked to LPS/GalN/DMSO intoxication [51,52,53]. MDA, a byproduct of lipid peroxidation, is frequently used to evaluate oxidative stress. In our study, CFZ effectively decreased LPS/GalN/DMSO-induced MDA levels. The findings show that CFZ protects the liver from LPS/GalN/DMSO-induced hepatic damage by reducing oxidative stress. This impact might be attributable to its ability to inhibit NF-кB activation. As a result, the current investigation demonstrated that CFZ may effectively lower the oxidative load during the inflammatory response to LPS/GalN/DMSO. GSH is widely recognized for its ability to protect cells from oxidative stress via non-enzymatic and enzymatic processes. At 6 h after LPS/GalN/DMSO injection, there was a substantial drop in hepatic GSH. LPS/GalN/DMSO-treated animals resulted in a significant depletion of cellular GSH. This might be connected to lipid peroxidation caused by LPS/GalN/DMSO [54,55]. At 6 h, mice treated with CFZ produced considerably more GSH than LPS/GalN/DMSO-treated animals. This might be related to its ability to reduce oxidative stress caused by LPS/GalN/DMSO by reducing free radical-mediated pathways.

## 5. Conclusions

**Figure 7:**
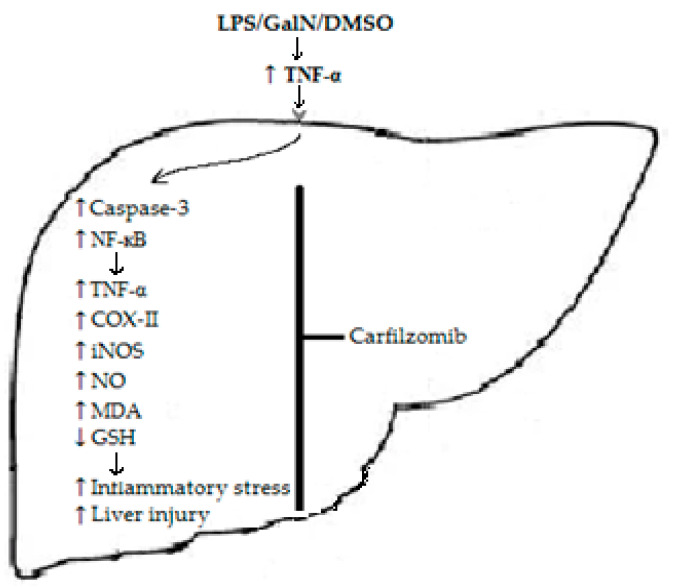
A schematic illustration of the possible mechanism(s) implicated in LPS/GalN/DMSO-caused hepatic cell death and its proposed points of interference with CFZ. ↑ = increase and ↓ = decrease.

Our findings show that administering various dosages of CFZ one hour after injecting LPS/GalN/DMSO resulted in a dose-dependent reduction in liver damage. CFZ can prevent the majority of the damage caused by LPS/GalN/DMSO in mice livers, and its protective effects are linked to the attenuation of apoptosis, the inhibition of the NF-кB pathway, the reduction in inflammatory mediator production, the suppression of oxidative and nitrosative stress, and the restoration of glutathione levels (Figure 7). The hepatoprotective characteristics of CFZ reported here make it a potential candidate for the treatment of inflammatory liver diseases. 

## Figures and Tables

**Table 1:** The primers used in RT-PCR.

Gene	GenBank Accession #	Annealing Temperature	Orientation	Sequences (5’ to 3’)
COX-II	NM_011198	60 °C	Forward Reverse	TGGTGCCTGGTCTGATGATG GTGGTAACCGCTCAGGTGTTG
iNOS	NM_010927	60 °C	Forward Reverse	CCTGGTACGGGCATTGCT GCTCATGCGGCCTCCTTT
GAPDH	NM_008084	60 °C	Forward Reverse	TGAAGCAGGCATCTGAGGG CGAAGGTGGAAGAGTGGGAG

## Data Availability

The authors confirm that all data underlying the findings are fully available without restriction.
